# ERCP management of cystic duct stones with a rare anatomic variant in octogenarian: a case report

**DOI:** 10.3389/fsurg.2026.1801270

**Published:** 2026-05-04

**Authors:** Yunlong Zhang, Shuai Zhang, Feng Li, Xiaoshuai Jing, Qi Liu, Muchuan Yu

**Affiliations:** Department of General Surgery, Jinan Zhangqiu District People’s Hospital, Jinan, China

**Keywords:** case report, cystic duct stones, endoscopic retrograde cholangiopancreatography, low and left-sided insertion of the cystic duct, oldest-old

## Abstract

**Background:**

Endoscopic retrograde cholangiopancreatography (ERCP) is a critical therapeutic modality for bile duct stones; however, cases of cystic duct stones (CDS) managed by ERCP alone are rarely reported. Herein, we present a rare case of an octogenarian patient with an anatomic variation consisting of low and left-sided insertion of the cystic duct (CD) in whom both bile duct stones and CDS were successfully treated using ERCP.

**Case presentation:**

An 82-year-old male presented to our hospital with an acute epigastric pain associated with fever and chills. Diagnostic imaging revealed multiple bile duct stones and CDS, along with an anatomical variant of the low and left-sided insertion of the CD. The patient declined to undergo laparoscopic cholecystectomy (LC) with laparoscopic common bile duct exploration (LCBDE) because of concerns about surgical risks. All stones were successfully removed via ERCP, with preservation of the gallbladder function. The patient experienced no postoperative complications, and no stone recurrence was detected during the 1-year follow-up period.

**Conclusion:**

This case demonstrates that an ERCP-only approach represents a safe and effective therapeutic strategy for patients with biliary anatomic variations complicated by complex stone disease, particularly in the oldest-old (≥80 years) patients who are ineligible for laparoscopic surgery. The postoperative recurrence rate of biliary stones remained high in this patient population. In such cases, a repeat ERCP is a viable and reasonable therapeutic option.

## Introduction

Cholelithiasis has a high prevalence among elderly populations (≥65 years). Biliary obstruction caused by stones can lead to rapidly progressing infections and systemic sepsis or even toxic shock if not intervened in time (1). For patients with concomitant gallbladder stones and common bile duct stones, the primary surgical options are laparoscopic cholecystectomy (LC) with laparoscopic common bile duct exploration (LCBDE), or ERCP followed by LC. The 2015 National Institutes of Health guidelines affirmed the efficacy of these two approaches (2). It should be noted that oldest-old patients (≥80 years) are at significantly increased risk of mortality during biliary tract surgery (3, 4). Endoscopic removal of both bile duct stones and CDS via ERCP is technically challenging, particularly in the presence of an anatomical variant of low and left-sided insertion of the CD.

## Case report

**Figure 1:**
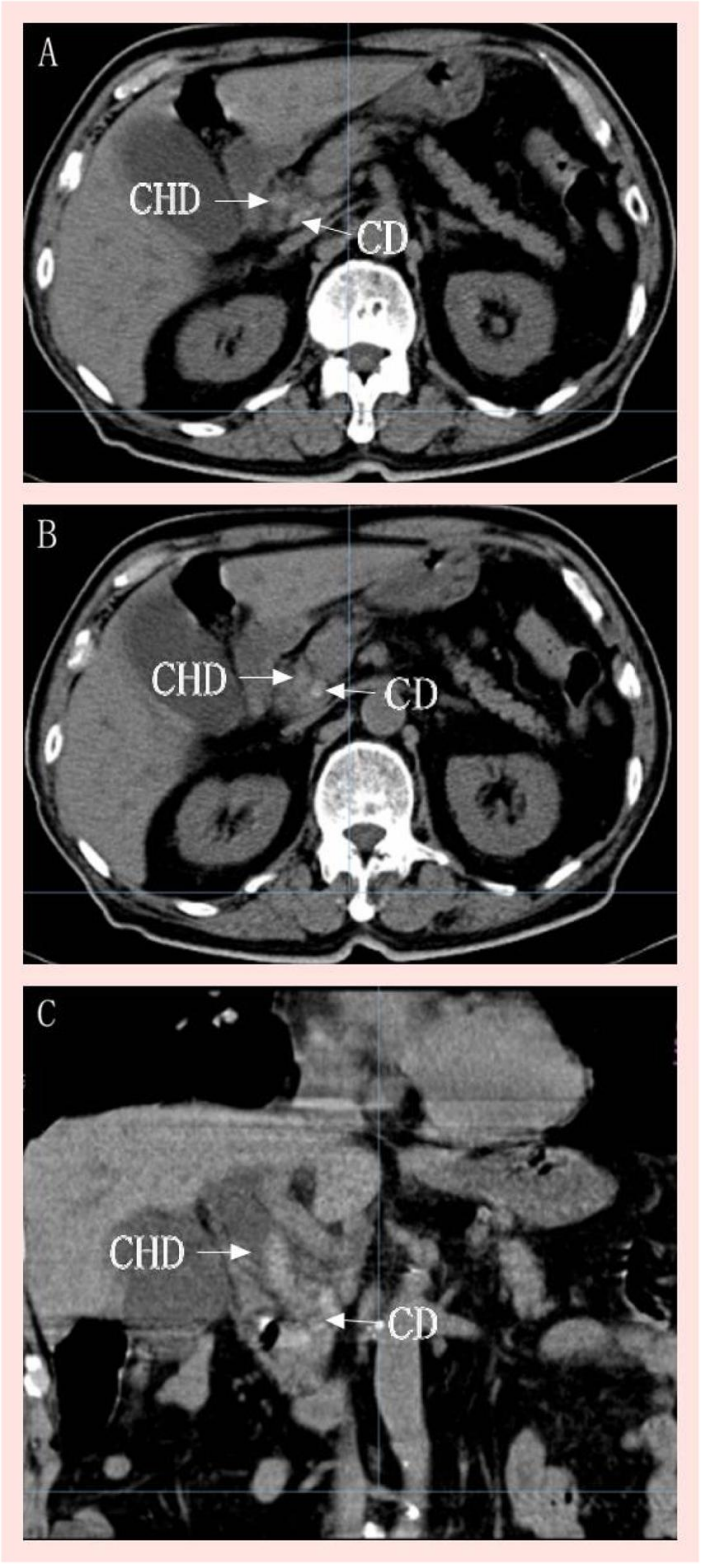
Preoperative upper abdominal CT: multiple stones in the bile duct and CD, with marked dilation of the extrahepatic bile duct; panels **(A)** and **(B)** demonstrate the CD coursing dorsally from the common hepatic duct to the left side, running parallel to the common hepatic duct. Panel **(C)** reveals the low confluence of the CD into the bile duct. CD, cystic duct; CHD, common hepatic duct.

An 82-year-old Chinese male presented to our hospital with a 2-day history of acute upper abdominal pain. Two days prior to admission, he developed sudden-onset, persistent colicky epigastric pain accompanied by vomiting of gastric contents, fever (38.5 °C), and chills. His past medical history was notable only for well-controlled hypertension, with no prior surgical history or other known comorbidities. His body mass index was 23.5 kg/m^2^. He denied smoking, alcohol consumption, or illicit drug use, and reported no family history of hereditary diseases or malignancy. Physical examination revealed mild icterus, significant tenderness in the right upper quadrant, and a positive Murphy's sign. Laboratory tests revealed leukocytosis (11.49 × 10^9^/L), cholestasis (total bilirubin 60.9 μmol/L, direct bilirubin 35.7 μmol/L), and significant transaminase elevation (alanine aminotransferase 340 U/L, aspartate aminotransferase 675 U/L). Abdominal computed tomography (CT) (Figure 1) revealed choledocholithiasis with biliary obstruction, cholecystitis, and an anatomical variant of CD inserted into the left lateral wall of the common bile duct. MRCP was not performed because the patient's hearing impairment and limited cooperation made it difficult for him to follow commands (e.g., prolonged supine positioning and breath-holding) required for MRI-based imaging. The final diagnoses were bile duct stones complicated by acute cholangitis, cystic duct stones with acute cholecystitis in the setting of an anatomic cystic duct variation, obstructive jaundice, and hypertension.

**Figure 2:**
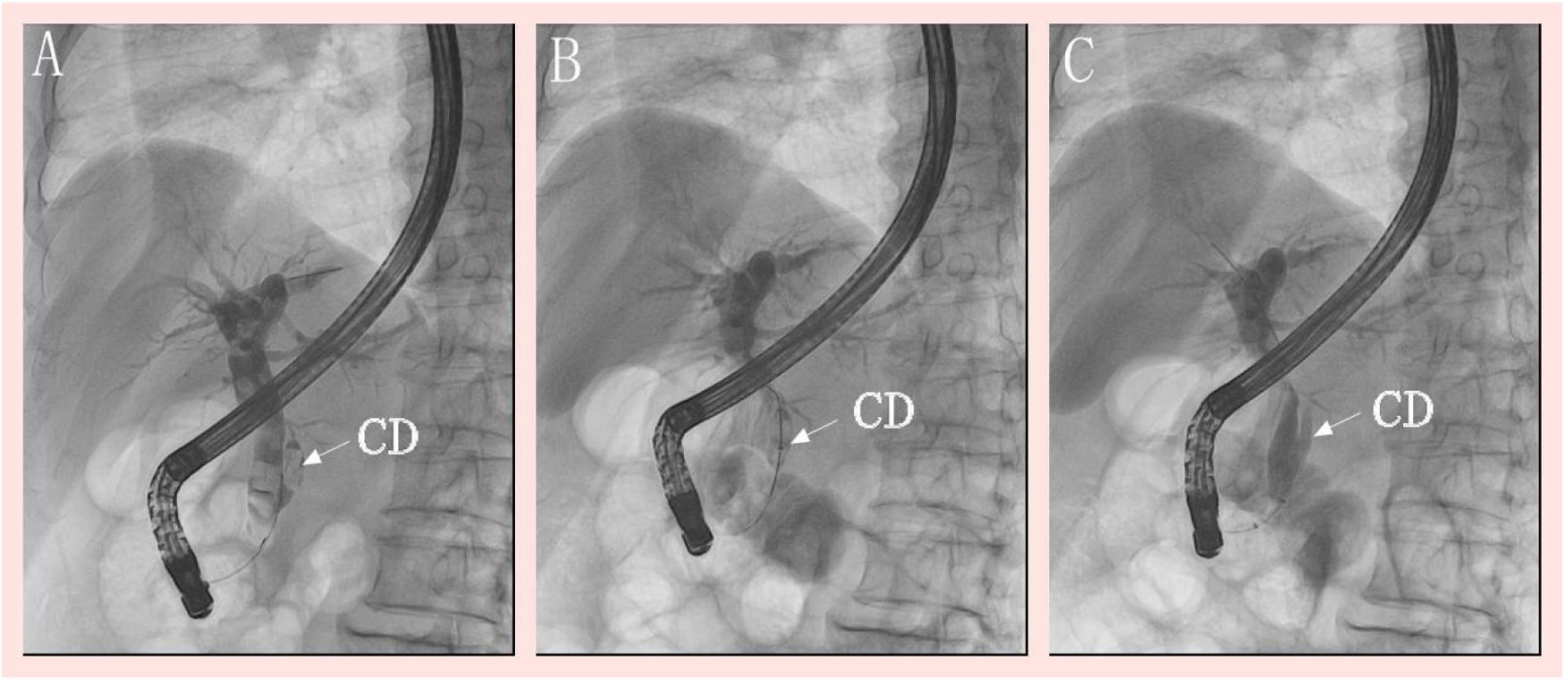
Endoscopic retrograde cholangiopancreatography: **(A)** left low confluence CD, multiple filling defects of bile duct and CD; **(B)** guide wire superselective inserted into the CD, the CDS were removed by a extraction balloons; **(C)** extraction balloon cholangiography confirmed no residual filling defects. CD, cystic duct; CDS, cystic duct stones.

Preoperative evaluation confirmed normal cardiopulmonary function without absolute contraindications to surgery. However, after we explained to the patient and his family that leaving the gallbladder *in situ* could predispose to recurrent biliary events, they declined cholecystectomy because of the patient's advanced age and their concerns about operative risk; therefore, a minimally invasive endoscopic approach was selected. On June 30, 2023, under intravenous general anesthesia, the patient underwent ERCP combined with endoscopic sphincterotomy, papillary balloon dilation, extraction of the bile duct and CDS, and endoscopic nasobiliary drainage. Cholangiography revealed dilation of the extrahepatic bile duct (approximately 13 mm) with multiple filling defects (largest diameter: 11 mm). The CD was visualized on the lower left side of the bile duct (approximately 15 mm from the papilla), containing filling defects. A minor incision in Oddi's sphincter was made at the 11 o'clock position of the papilla, followed by balloon dilation (12 mm). Stones in the extrahepatic bile duct were sequentially removed. Biliary cannulation was achieved after three attempts, with inadvertent pancreatic duct cannulation occurring twice. A hydrophilic zebra guidewire was then superselectively inserted into the CD, and cholangiography identified three filling defects (maximum diameter: 8 mm). An extraction balloon was used to dislodge the stones into the extrahepatic bile duct, from which they were subsequently extracted (Figure 2), and a nasobiliary drain was placed. The total procedure time was 102 min; no lithotripsy was required, and all stones were removed intact.

**Figure 3:**
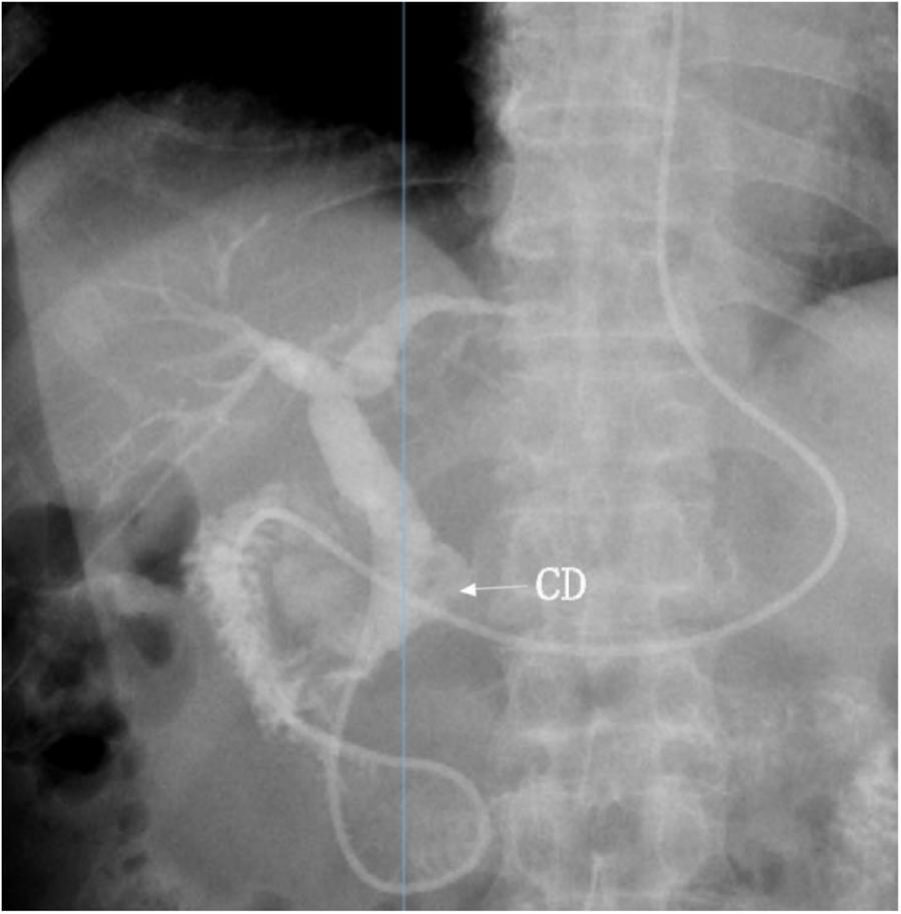
Nasobiliary cholangiography: no filling defect was found in the gallbladder, CD, intrahepatic, and extrahepatic bile ducts. CD, cystic duct.

No complications such as pancreatitis or gastrointestinal bleeding occurred. By postoperative day 3, a short fluoroscopic nasobiliary cholangiography was completed with minimal cooperation, demonstrating clear visualization of the gallbladder, cystic duct, and intra-/extrahepatic bile ducts without residual stones (Figure 3).

The patient was kept nil per os for the first 24 h postoperatively and then gradually advanced to a liquid diet. On postoperative day 3, after nasobiliary cholangiography confirmed complete stone clearance, the nasobiliary drain was removed and a regular diet was resumed; the patient was discharged in stable condition on postoperative day 4. During the 1-year follow-up, the patient remained asymptomatic with no abdominal pain, fever, or jaundice, and MRCP at 12 months showed no evidence of stone recurrence.

## Discussion

ERCP is one of the highest-risk endoscopic procedures, with a reported post-ERCP pancreatitis rate as high as 9.7%, and is also among the most technically demanding interventions (5). Numerous studies have demonstrated that there is no significant difference in technical success rates or procedure-related complications between oldest-old patients and younger patients undergoing ERCP (4, 6). Notably, in the study by Frank et al. (7), patients aged over 80 years exhibited a significantly lower incidence of post-ERCP pancreatitis than those under 80 years of age (0.9% vs. 5.3%; *P* < 0.05) (4). Therefore, ERCP remains a safe therapeutic option, even in oldest-old patients.

The concomitant endoscopic management of choledocholithiasis and CDS via ERCP is technically challenging. He et al. (8) first reported six successful cases of ERCP-based management of cholecystolithiasis combined with choledocholithiasis. Notably, none of these cases involved left-sided insertion of the CD, which is considered one of the most technically difficult anatomic variations. Subsequently, Pawa et al. (9) reported 21 similar cases, of which 15 were managed with mother-baby choledochoscopy plus electrohydraulic lithotripsy. It is important to highlight that all patients with left-sided insertion of the CD in their series required this combined technique. However, this approach carries a higher risk of biliary tract injury and potential residual microlithiasis, which may increase the recurrence rates and warrant cautious clinical application. During our ERCP procedure, when the sphincterotome was advanced into the common channel, the tip orientation was adjusted to align with the course of the CD, thus facilitating guidewire insertion into the dilated CD. This maneuver is a critical factor in procedural success. Compared with laparoscopic surgery, ERCP offers advantages such as scarless natural orifice access, preservation of gallbladder function, reduced rates of biliary injury and other procedure-related complications, and better patient tolerance (8). These benefits make ERCP a safer and more effective option for the oldest-old patients.

Anatomical variations in CD are the most common among all variations in the biliary system. In this case, the CD exhibited an extremely low confluence near the Vater ampulla, with a dorsal looping course around the common bile duct before merging from the left side. The reported incidence of low and left-sided insertion of the CD variant is as low as 0.2% (10). Relevant studies applied Poiseuille's law to explain that a longer CD impedes bile drainage (11). Additionally, a confluence close to the sphincter of Oddi may partially impair contractile function (12).

Sphincterotomy of Oddi's sphincter increases the risk of enteric contents and bacterial reflux into the biliary tract. Notably, patients over 65 years of age exhibited a recurrence rate as high as 30%, with 86.4% of recurrent cases occurring in this age group (13). Therefore, this patient was at a significantly increased risk of stone recurrence. If the approach of LC plus LCBDE were chosen, its advantage would have been the preservation of the sphincter of Oddi's function. However, this technique poses challenges for achieving complete CD resection. In anatomical variations such as this, dissection of the distal CD carries the risk of injury to the bile duct and pancreas, and complete removal of the CDS is often not feasible. Residual stones in CD may lead to post-cholecystectomy syndrome (14), creating a therapeutic dilemma. We propose that for such patients, a staged approach-performing ERCP for complete stone clearance followed by LC is a viable strategy, provided the patient is clinically stable and written informed consent for publication was obtained from the family. During LC, meticulous dissection of the cystic duct and its transection at the lowest anatomically safe level are crucial for minimizing the risk of recurrence. Although this strategy requires advanced surgical skills, it offers significant long-term benefits for carefully selected patients. However, for cases of high-risk recurrence, repeat ERCP is a reasonable treatment option (15, 16).

## Conclusion

This case demonstrates that an ERCP-only approach represents a safe and effective therapeutic strategy for patients with biliary anatomic variations complicated by complex stone disease, particularly in the oldest-old patients who are ineligible for laparoscopic surgery. Furthermore, ERCP offers distinct advantages over conventional surgical interventions, including minimal invasiveness and preservation of sphincter function where appropriate. However, certain limitations persist, most notably the technical challenges associated with achieving superselective cannulation of CD. Additionally, the postoperative recurrence rate of biliary stones remains high in this patient population. In such cases, a repeat ERCP is a viable and reasonable therapeutic option. This approach underscores the importance of endoscopic expertise and tailored patient management of complex biliary disorders.

## Data Availability

The original contributions presented in the study are included in the article/Supplementary Material, further inquiries can be directed to the corresponding author.
